# PPVED: A machine learning tool for predicting the effect of single amino acid substitution on protein function in plants

**DOI:** 10.1111/pbi.13823

**Published:** 2022-04-27

**Authors:** Xiangjian Gou, Xuanjun Feng, Haoran Shi, Tingting Guo, Rongqian Xie, Yaxi Liu, Qi Wang, Hongxiang Li, Banglie Yang, Lixue Chen, Yanli Lu

**Affiliations:** ^1^ State Key Laboratory of Crop Gene Exploration and Utilization in Southwest China Wenjiang Sichuan China; ^2^ 12529 Maize Research Institute Sichuan Agricultural University Wenjiang Sichuan China; ^3^ Chengdu Academy of Agricultural and Forestry Sciences Wenjiang Sichuan China; ^4^ 47895 National Key Laboratory of Crop Genetic Improvement Huazhong Agricultural University Wuhan Hubei China; ^5^ 12529 Triticeae Research Institute Sichuan Agricultural University Wenjiang Sichuan China; ^6^ 12529 College of Information Engineering Sichuan Agricultural University Ya’an Sichuan China

**Keywords:** single amino acid substitution, protein, functional effect, machine learning

## Abstract

Single amino acid substitution (SAAS) produces the most common variant of protein function change under physiological conditions. As the number of SAAS events in plants has increased exponentially, an effective prediction tool is required to help identify and distinguish functional SAASs from the whole genome as either potentially causal traits or as variants. Here, we constructed a plant SAAS database that stores 12 865 SAASs in 6172 proteins and developed a tool called Plant Protein Variation Effect Detector (PPVED) that predicts the effect of SAASs on protein function in plants. PPVED achieved an 87% predictive accuracy when applied to plant SAASs, an accuracy that was much higher than those from six human database software: SIFT, PROVEAN, PANTHER‐PSEP, PhD‐SNP, PolyPhen‐2, and MutPred2. The predictive effect of six SAASs from three proteins in *Arabidopsis* and maize was validated with wet lab experiments, of which five substitution sites were accurately predicted. PPVED could facilitate the identification and characterization of genetic variants that explain observed phenotype variations in plants, contributing to solutions for challenges in functional genomics and systems biology. PPVED can be accessed under a CC‐BY (4.0) license via http://www.ppved.org.cn.

## Introduction

Single amino acid substitutions (SAASs) are usually caused by single‐nucleotide variants in the coding region of a gene (Care *et al*., 2007; Ng and Henikoff, 2006; Wang *et al*., 2012). Some SAASs can affect normal protein function (defined as functional SAASs), leading to obvious physiological or morphological changes in plants (Li *et al*., 2012; Wang *et al*., 2016; Xu *et al*., 2018). Large‐scale diversity investigations of the various human genomes, including malignant tumour genomes, reveal that SAASs are the most encountered variants (Lek *et al*., 2016). As a large quantity of SAASs is distributed throughout the whole genome, it is challenging to identify functional variants from all the substitutions, and distinguish large effect alterations with other variant versions at the same position. However, tabulating the effect of SAASs on specific proteins is a necessity for annotating gene (and protein) functions and interactions, and provides insights into the molecular basis of biological activity and molecular mechanisms of complex traits (Kono *et al*., 2018; Kovalev *et al*., 2018; Wang *et al*., 2012).

Traditional experimental methods can accurately assess the effect of SAASs on protein function; however, these methods are time‐consuming, resource‐intensive, and difficult to manipulate (Ng and Henikoff, 2006). Moreover, data accumulation from whole‐genome sequencing and resequencing analysis in projects, such as de novel assemblies for a pan‐genome in rice (Zhao *et al*., 2018), maize (Hufford *et al*., 2021), sorghum (Tao *et al*., 2021), and the *Arabidopsis* 1001 genome project (Carlos *et al*., 2016), has resulted in a substantial increase of SAAS numbers, which further renders these traditional methods ineffective. To annotate the SAASs in a high‐throughput manner, one potential avenue is the use of computational methods to predict the effect of SAASs on protein function, prioritizing functional SAASs for subsequent experimental assessment (Kovalev *et al*., 2018; Ng and Henikoff, 2006).

Many software programs have been developed to predict the effect of SAASs on protein function in humans (Ng and Henikoff, 2001; Stone and Sidow, 2005; Capriotti *et al*., 2006; Chun and Fay, 2009; Adzhubei *et al*., 2010; Choi *et al*., 2012; Wang *et al*., 2012; Niroula *et al*., 2015; Hecht *et al*., 2015; Quang *et al*., 2015; Tang and Thomas, 2016; Ioannidis *et al*., 2016; Alirezaie *et al*., 2018; Chennen *et al*., 2020; Pejaver *et al*., 2020; Takeda *et al*., 2020). These programs are linked to molecular variant databases, such as dbSNP (Sherry *et al*., 2001), ClinVar (Landrum *et al*., 2018), UniProt (Yip *et al*., 2010), HGMD (Stenson *et al*., 2017), OMIM (Amberger *et al*., 2014), SNPdbe (Schaefer *et al*., 2012), VariBench (Nair and Vihinen, 2013), and VariSNP (Schaafsma and Vihinen, 2015). Based on different prediction principles, existing software can be grouped into three categories: calculation of the conservative index of amino acids by aligning the query protein with the target protein library; establishment of a machine learning model with inputs of protein sequences, structures, and post‐translational modifications; construction of a hybrid method with precalculated scores of SAASs (recorded in the dbNSFP database) (Liu *et al*., 2015) as input features for machine learning algorithms.

Few studies have focused on developing a method or pipeline for predicting SAASs effect on protein function in plants owing to the lack of plant SAAS resources collected from molecular experiments (Kovalev *et al*., 2018). Although some software (e.g., SIFT, MAPP, and PROVEAN), developed based on human SAASs, have been applied to predict altered protein function in plants (Feiz *et al*., 2009; Günther and Schmid, 2010; Chen *et al*., 2012; Mezmouk and Ross‐Ibarra, 2013; Kuppu *et al*., 2015; Yang *et al*., 2017; Krasileva *et al*., 2017; Kim *et al*., 2021), this distant cross‐species application has common undesirable aspects, such as low predictive accuracy and contradictory prediction results. Thus, the robustness of this approach cannot be maintained when applied to SAAS detection in plants (Feiz *et al*., 2009; Kono *et al*., 2018).

**Figure 1:**
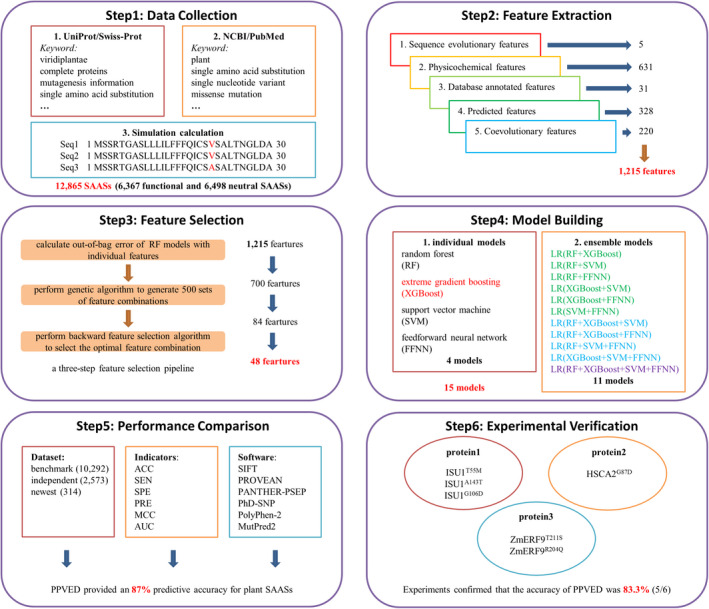
Overview of this study to develop PPVED and use it to predict the effect of SAASs on protein function in plants. To develop PPVED, we first collected plant SAAS dataset from three sources, including: UniProt/Swiss‐Prot, NCBI/PubMed, and simulation calculation based on multiple sequence alignments. Second, we comprehensively collected the features that characterize SAASs from five categories. Third, to reduce the dimension of features and avoid model overfitting, we proposed a three‐step feature selection pipeline and used this pipeline for feature selection. Then, we built 15 machine learning models, including four individual models and 11 ensemble models based on 4 learning algorithms by using the selected features. Moreover, we further selected six popular existing software developed based on human SAASs for performance comparison with PPVED. Finally, the prediction accuracy of PPVED was proved through experiments on three proteins.

Here, we designed a novel Plant Protein Variation Effect Detector (PPVED) that predicts the effect of SAASs on protein function in plants through accumulated experimental information, (re)sequencing data, and advanced analytical algorithms (Figure 1). PPVED is linked to a plant SAAS database, manually constructed with multiple resources having experimental evidence. After data processing, we built 4 individual and 11 ensemble models for classifying functional and neutral SAASs using random forest (RF), extreme gradient boosting (XGBoost), support vector machine (SVM), and feedforward neural network (FFNN). Among these models, XGBoost performed best in the model evaluation process and was selected as the core algorithm in PPVED. To validate the predictive accuracy of PPVED, we used three different datasets of SAASs, and compared PPVED with six existing human database software. The results demonstrated the high accuracy of PPVED in predicting the effect of SAASs on protein function.

## Results

### A total of 12 865 plant SAASs were collected from resources with experimental evidence

A plant SAAS database with 12 865 SAASs in 6172 proteins was constructed with three different sources: UniProt/Swiss‐Prot, NCBI/PubMed, and simulation calculation based on multiple sequence alignments (Figure 1). The numbers of functional and neutral SAASs identified were 6367 and 6498, respectively, indicating balanced data gathering. For model learning and inference, we split the overall dataset into two subsets with random sampling: 80% in the benchmark dataset for model learning versus 20% in the independent dataset for model inference. The benchmark dataset was used for subsequent feature extraction, feature selection, and model building, while the independent dataset was used to assess the generalization ability of the model. The proportions of subcategories (functional and neutral) were generally balanced in each subset (benchmark or independent).

### Forty‐eight informative features were selected from 1215 candidate features

A total of 1215 features, extracted through various computational methods, were used as input variables for predictive model development. These features were classified into five categories: sequence evolutionary features (5), physicochemical features (631), database annotated features (31), predicted features (328), and coevolutionary features (220). To reduce the feature dimensions and avoid overfitting, 48 features from 1215 candidates were selected using the three‐step feature selection pipeline proposed in this study (see Table S1 for the meaning of each feature). We evaluated the model performance changes before and after feature selection, and the results indicated the performance after feature selection was significantly higher than that before feature selection, demonstrating the utility of the feature selection pipeline (Figure S1). Moreover, the efficiency of model building also increased by nearly 30 times after feature selection. We detected that at least two features were selected from each of the five categories (Table S1). Sequence evolutionary features were retained in the largest proportion, although only five items were initially collected (2/5, 40%). Physicochemical features, which accounted for the largest part of all variables, were kept as informative features in the lowest proportion (18/631, 2.85%).

**Figure 2:**
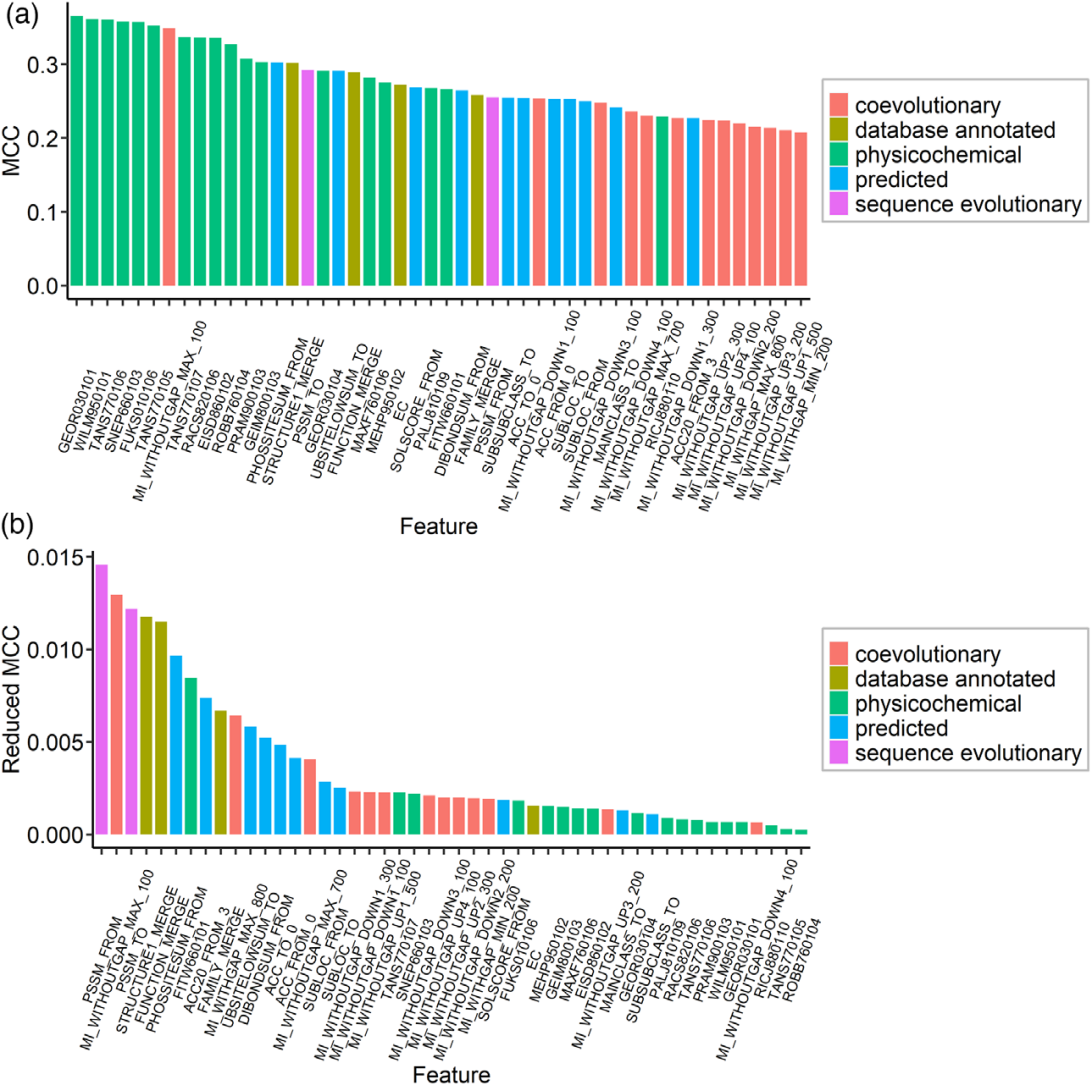
Importance evaluation of 48 features. (a) Performance of training models by using only a single feature. (b) The performance loss that removed the single feature and used the remaining features to train the models. A one‐tailed *t*‐test showed that the reduction in performance was significant upon removing the single feature (*P* < 0.05). The above performance was evaluated and represented by Matthew’s correlation coefficient (MCC).

To determine the importance of each of the 48 features in the predictive models, we adopted two strategies: keeping only a single feature in the model or removing the single feature from the full model that includes all 48 features. The results showed that the importance indicator, the Matthew’s correlation coefficient (MCC), was larger than 0.2 for all features, demonstrating the significance of selected variables (Figure 2a). Notably, almost all physicochemical features had better MCC performance than the other features (Figure 2a). Additionally, we found that when removed from models, the single sequence evolutionary feature caused the largest performance loss (Figure 2b). In the case of removal of the features PSSM_FROM (position‐specific score of wildtype amino acid) and PSSM_TO (position‐specific score of mutant amino acid), MCC values decreased by 0.0146 and 0.0122, respectively. A one‐tailed *t*‐test revealed that the reduction of MCC values was statistically significant (*P *< 0.05). Moreover, we also found that the removal of all predicted features caused the most severe performance loss compared with removing other features categories. (Figure S2). Overall, the 48 features selected from the 1215 candidates were examined based on feature importance analyses; they were all found to be essential for building a predictive model. These features can improve model performance and reduce computational cost as compared with the full model.

### Model evaluation selected the XGBoost algorithm for PPVED

With 48 informative features, we built 15 machine learning models based on 4 types of learning algorithms: RF, XGBoost, SVM, and FFNN, which included 4 individual models and 11 ensemble models. The prediction results of ensemble models were synthesized from multiple individual models using the stacking method. The global performance of predictive models was evaluated based on six indicators: sensitivity (SEN), specificity (SPE), precision (PRE), accuracy (ACC), MCC, and area under the curve (AUC). After applying these models in the benchmark dataset with 10,292 SAASs (Table 1) and the independent dataset with 2,573 SAASs (Table 2), we found that the XGBoost‐based model had the best global performance compared with the other models. Although there were some benefits, the ensemble models did not predict results that were better than that of individual models. Specifically, when applied to the benchmark dataset, the XGBoost‐based model generated the largest values of MCC (0.744), ACC (0.872), and AUC (0.940), and comparable results for SEN (0.886), SPE (0.857), and PRE (0.859) (Table 1). Using the independent dataset, the XGBoost‐based model generated similar prediction results, demonstrating a robust generalization ability (Table 2). Owing to the good model learning and evaluation performance, the XGBoost algorithm was selected for PPVED to predict the effect of SAASs on protein function for prioritizing the functional variants.

**Table 1:** Performance comparison of 15 models in the benchmark dataset

Algorithm	MCC	ACC	SEN	SPE	PRE	AUC
RF	0.731	0.865	0.890	0.840	0.845	0.929
XGBoost	0.744	0.872	0.886	0.857	0.859	0.940
SVM	0.663	0.831	0.852	0.810	0.815	0.902
FFNN	0.648	0.824	0.837	0.811	0.813	0.891
LR(RF+XGBoost)	0.737	0.868	0.881	0.855	0.857	0.935
LR(RF+SVM)	0.721	0.860	0.873	0.848	0.849	0.929
LR(RF+FFNN)	0.722	0.861	0.874	0.848	0.849	0.929
LR(XGBoost+SVM)	0.737	0.869	0.881	0.856	0.857	0.934
LR(XGBoost+FFNN)	0.741	0.870	0.881	0.860	0.860	0.935
LR(SVM+FFNN)	0.663	0.831	0.846	0.816	0.818	0.903
LR(RF+XGBoost+SVM)	0.737	0.868	0.881	0.856	0.857	0.935
LR(RF+XGBoost+FFNN)	0.735	0.868	0.879	0.856	0.857	0.935
LR(RF+SVM+FFNN)	0.721	0.860	0.873	0.848	0.849	0.929
LR(XGBoost+SVM+FFNN)	0.738	0.869	0.881	0.856	0.857	0.934
LR(RF+XGBoost+SVM+FFNN)	0.736	0.868	0.879	0.856	0.857	0.935

ACC, accuracy; AUC, area under the curve of the receiver operating characteristic; FFNN, feedforward neural network; LR, logistic regression; MCC, Matthew’s correlation coefficient; PRE, precision; RF, random forest; SEN, sensitivity; SPE, specificity; SVM, support vector machine; XGBoost, extreme gradient boosting.

**Table 2:** Performance comparison of 15 models in the independent dataset

Algorithm	MCC	ACC	SEN	SPE	PRE	AUC
RF	0.687	0.843	0.871	0.815	0.822	0.916
XGBoost	0.712	0.856	0.874	0.838	0.841	0.931
SVM	0.632	0.815	0.854	0.777	0.789	0.889
FFNN	0.627	0.813	0.844	0.782	0.791	0.889
LR(RF+XGBoost)	0.710	0.855	0.869	0.842	0.843	0.924
LR(RF+SVM)	0.677	0.838	0.856	0.821	0.824	0.916
LR(RF+FFNN)	0.679	0.839	0.855	0.824	0.826	0.916
LR(XGBoost+SVM)	0.714	0.857	0.871	0.842	0.844	0.925
LR(XGBoost+FFNN)	0.710	0.855	0.870	0.841	0.842	0.926
LR(SVM+FFNN)	0.635	0.817	0.844	0.791	0.798	0.893
LR(RF+XGBoost+SVM)	0.710	0.855	0.870	0.840	0.842	0.924
LR(RF+XGBoost+FFNN)	0.709	0.854	0.868	0.841	0.842	0.924
LR(RF+SVM+FFNN)	0.679	0.839	0.856	0.822	0.825	0.916
LR(XGBoost+SVM+FFNN)	0.714	0.857	0.871	0.842	0.844	0.925
LR(RF+XGBoost+SVM+FFNN)	0.708	0.854	0.867	0.841	0.842	0.924

ACC, accuracy; AUC, area under the curve of the receiver operating characteristic; FFNN, feedforward neural network; LR, logistic regression.; MCC, Matthew’s correlation coefficient; PRE, precision; RF, random forest; SEN, sensitivity; SPE, specificity; SVM, support vector machine; XGBoost, extreme gradient boosting.

### PPVED surpassed existing software when applied to SAASs in plants

**Figure 3:**
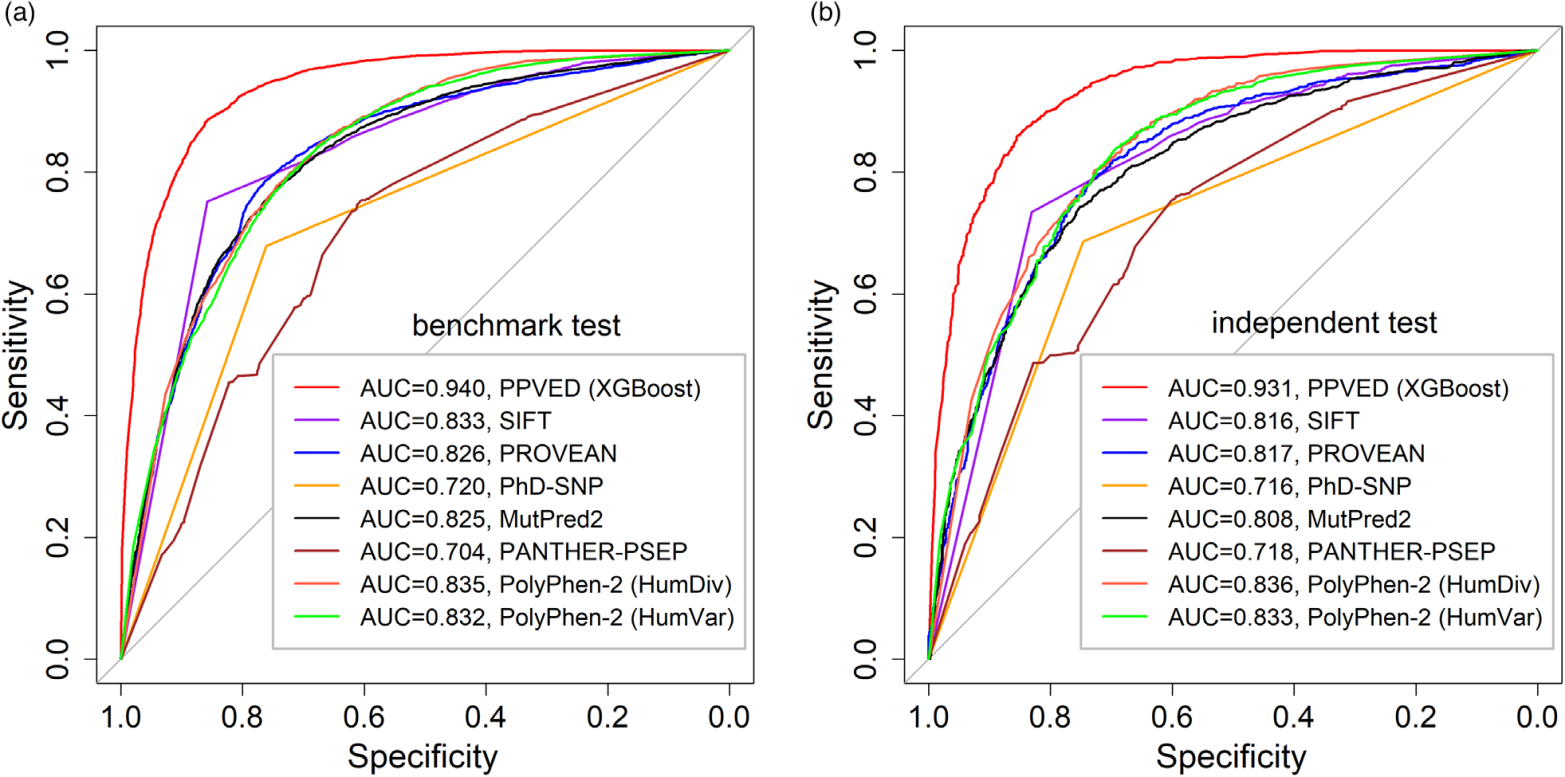
Receiver operating characteristic (ROC) curves of six popular existing software and PPVED in benchmark dataset and independent dataset, respectively. (a) ROC curves under benchmark dataset. (b) ROC curves under independent dataset. The two models provided by PolyPhen‐2, including HumDiv and HumVar, were considered. The area under the curve (AUC) of the ROC curve was also showed in the figure.

We compared the performance of PPVED with the six most‐used software (SIFT, PROVEAN, PANTHER‐PSEP, PhD‐SNP, PolyPhen‐2, and MutPred2) that link to the human SAAS dataset. Six indicators of global performance for each software are listed in Table 3 for the benchmark dataset and in Table 4 for the independent dataset. We further visualized the model performance by plotting the receiver operating characteristic (ROC) curve represented by the area under the ROC curve (AUC; Figure 3). The AUC values were remarkably high for PPVED, moderate for PROVEAN, SIFT, PolyPhen‐2, and MutPred2, and low for PhD‐SNP and PANTHER‐PSEP in both datasets, demonstrating the good predictive performance of PPVED. Besides AUC, other indicators also manifested the advantages of PPVED; for example, the ACC values were 10% higher than those from the second‐best software PROVEAN. The high values of all indicators of model performance suggest that PPVED is a powerful tool for accurately separating functional SAASs from neutral ones in plant datasets.

**Table 3:** Performance comparison of six existing software and PPVED under benchmark dataset

Software	MCC	ACC	SEN	SPE	PRE	AUC
SIFT*	0.475	0.726	0.873	0.581	0.671	0.833
PROVEAN	0.547	0.773	0.774	0.772	0.769	0.826
PANTHER‐PSEP^†^	0.356	0.681	0.756	0.594	0.681	0.704
PhD‐SNP	0.442	0.720	0.679	0.761	0.736	0.720
PolyPhen‐2 (HumDiv)^‡^	0.527	0.762	0.868	0.642	0.733	0.835
PolyPhen‐2 (HumVar)^‡^	0.525	0.763	0.824	0.695	0.754	0.832
MutPred2	0.459	0.717	0.544	0.886	0.824	0.825
PPVED (XGBoost)	0.744	0.872	0.886	0.857	0.859	0.940

ACC, accuracy; AUC, area under the curve of the receiver operating characteristic; MCC, Matthew’s correlation coefficient; PRE, precision; SEN, sensitivity; SPE, specificity.

* For SIFT, 21 SAASs in the benchmark dataset cannot be predicted.

^†^ For PANTHER‐PSEP, 5632 SAASs cannot be predicted.

^‡^ For PolyPhen‐2, 778 SAASs cannot be predicted.

**Table 4:** Performance comparison of six existing software and PPVED under independent dataset

Software	MCC	ACC	SEN	SPE	PRE	AUC
SIFT*	0.462	0.718	0.876	0.564	0.663	0.816
PROVEAN	0.512	0.756	0.761	0.751	0.749	0.817
PANTHER‐PSEP^†^	0.346	0.676	0.765	0.574	0.675	0.718
PhD‐SNP	0.433	0.716	0.686	0.746	0.726	0.716
PolyPhen‐2 (HumDiv)^‡^	0.534	0.766	0.866	0.653	0.738	0.836
PolyPhen‐2 (HumVar)^‡^	0.532	0.767	0.820	0.708	0.760	0.833
MutPred2	0.432	0.704	0.529	0.876	0.807	0.808
PPVED (XGBoost)	0.712	0.856	0.874	0.838	0.841	0.931

ACC, accuracy; AUC, area under the curve of the receiver operating characteristic; MCC, Matthew’s correlation coefficient; PRE, precision; SEN, sensitivity; SPE, specificity.

* For SIFT, 8 SAASs in the independent dataset cannot be predicted.

^†^ For PANTHER‐PSEP, 1415 SAASs cannot be predicted.

^‡^ For PolyPhen‐2, 181 SAASs cannot be predicted.

### Newly collected SAASs further validated the PPVED generalization ability

More real genetic variants are required to further validate the proposed model of PPVED. Therefore, we performed an assessment of the generalization ability of PPVED using two different datasets. First, we collected 314 functional SAASs that were newly added to the UniProt/Swiss‐Prot knowledge base. As none of these SAASs were included in either the benchmark or the independent datasets, they can be considered as another testing set for validating the capability of PPVED to identify functional SAASs. We generated the input features for these new SAASs and predicted their effect on protein function. The results showed that 274 out of 314 were accurately predicted, a promising outcome demonstrating 0.873 predictive accuracy (Table S2). As expected, the prediction results were highly consistent with that shown in the independent dataset (Table 4, SEN = 0.874), thus demonstrating the capability of PPVED to identify functional variants. Specifically, four SAASs (R34A, S94A, W96A, and E100A) on the disease resistance protein RUN1 (UniProt accession number: V9M398) in *Vitis rotundifolia* were predicted as functional with the protein being reported to affect NAD^+^ cleavage activity (Horsefield *et al*., 2019). Moreover, two SAASs (K659E and D773L) on leucine‐rich repeat receptor protein kinase HPCA1 (UniProt accession number: Q8GZ99) in *Arabidopsis* were accurately predicted and reported to be responsible for catalytic activity loss (Wu *et al*., 2020).

Additionally, to validate the performance of PPVED for identifying neutral variants, 1515 neutral SAASs curated by a previous study (Kono *et al*., 2018) were tested using PPVED. These neutral SAASs have been adopted by another study (Kovalev *et al*., 2018) and are therefore representative. The results indicated that 1262 out of 1515 were accurately predicted, and the prediction accuracy was approximately 0.833 (Table S3). Similarly, the prediction results were consistent with that shown in the independent dataset (Table 4, SPE = 0.838). Overall, two additional datasets (functional and neutral SAASs) consistently demonstrated the generalization ability of PPVED.

### Predictive ability of PPVED experimentally validated in three proteins

The short and swollen root 1 (*SSR1*) gene encodes a mitochondrial protein and is involved in maintaining the mitochondrial electron transport chain function in *Arabidopsis* (Han *et al*., 2021; Zhang *et al*., 2015). However, the mechanism of *SSR1* in regulating these biological processes remains unclear. To detect suppressors of the knockout mutant *ssr1‐2*, we focused on protein candidates that may function to mask the short root phenotype of *ssr1‐*2 and used PPVED to predict the functional SAASs for each candidate. Two mitochondrial proteins known to participate in mitochondrial iron‐sulphur (Fe‐S) cluster biosynthesis (Roche *et al*., 2013), HSCA2 and ISU1, were considered as candidates for suppressor proteins by super bulked‐segregant analysis. Out of four SAASs in proteins HSCA2 and ISU1, three mutational sites (ISU1^A143T^, ISU1^G106D^, and HSCA2^G87D^) were predicted to be functional, and one (ISU1^T55 M^) was predicted to be neutral (Table 5).

**Table 5:** The prediction results of three wet lab experiments tested proteins

Protein	SAAS	Predicted score	Predicted class*	Observed class
ISU1^T55M^	T55M	0.244	Neutral	Functional
ISU1^A143T^	A143T	0.964	Functional	Functional
ISU1^G106D^	G106D	0.731	Functional	Functional
HSCA2^G87D^	G87D	0.999	Functional	Functional
ZmERF9^T211S^	T211S	0.018	Neutral	Neutral
ZmERF9^R204Q^	R204Q	0.001	Neutral	Neutral

* When Predicted score ≥ 0.5, Predicted class is predicted to be functional, and when Predicted score <0.5, Predicted class is predicted to be neutral (an explanation of why 0.5 was used as the threshold is shown in Figure S4).

**Figure 4:**
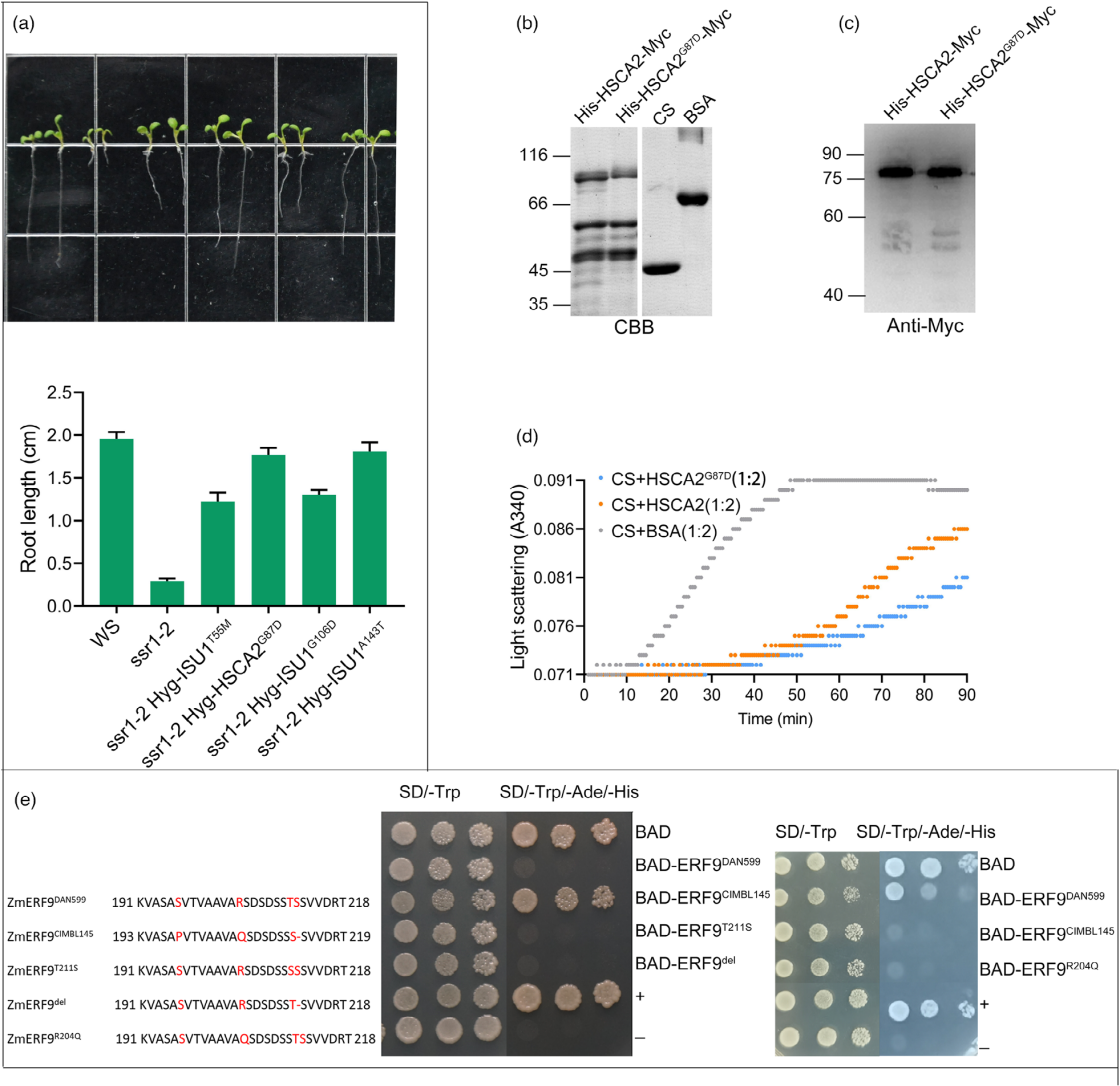
The predictive accuracy of PPVED in three proteins. (a) Four SAAS mutants of ISU1 and HSCA2 were individually introduced in *ssr1‐2* mutant, and the short root phenotype of *ssr1‐2* was remarkably rescued. WS is a wildtype ecotype of *Arabidopsis*. *ssr1‐2* is a T‐DNA inserted mutant containing the wildtype *ISU1* and *HSCA2* genes. The top panel is the representative seedlings. The bottom panel is the statistical results of root length. More than 30 seedlings were measured for each sample. (b) Purified His‐HSCA2‐Myc, His‐HSCA2^G87D^‐Myc, and commercial citrate synthase (CS) and bovine serum albumin (BSA) were isolated by SDS‐PAGE gel electrophoresis and stained with coomassie bright blue (CBB). (c) Detecting purified His‐HSCA2‐Myc and His‐HSCA2^G87D^‐Myc by western blot using anti‐Myc antibody. (d) Heat‐induced aggregation of CS was performed at 45°C for 90 min and monitored by increased light scattering at 340 nm. The molecular ratios of CS to tested proteins are 1:2. BSA was used as control sample. (e) The transcriptional inhibitory activity of different mutant sites was tested by yeast one‐hybrid. Different ZmERF9 was fused with Gal4‐AD and cloned into pGBKT7 to fuse with Gal4‐BD. The yeast cells harbouring indicated construct were grown on nonselective (SD/‐Trp) and selective (SD/‐Trp/‐Ade/‐His) medium to test the transcriptional inhibitory activity. Cells were diluted in three concentrations from left to right. Fused Gal4‐BD and Gal4‐AD protein (BAD) is a transcriptional activator. A pGBKT7‐53 and pGADT7‐T combination was used as a positive control (+). A pGBKT7‐Lam and pGADT7‐T combination was used as a negative control (‐). Partial amino acid sequence and mutant sites were showed in left panel.

We conducted a wet lab experiment that introduces the mutant gene in *ssr1‐2* to validate the effect of these four mutational sites. The results proved that three of the four sites were predicted correctly by PPVED, with ISU1^A143T^, ISU1^G106D^, and HSCA2^G87D^ displaying significant suppression of the short root phenotype of *ssr1‐2* (Figure 4a). We further confirmed protein activity change, exemplified by chaperone activity of HSCA2 (Leaden *et al*., 2014). To conduct this experiment, we first expressed *HSCA2* and *HSCA2^G87D^* in frame with 6× Histidine and 3× Myc in *Escherichia coli* (Figure 4b) and detected purified His‐HSCA2‐Myc and His‐HSCA2^G87D^‐Myc proteins by western blot (Figure 4c). Then, the purified proteins were used to test general chaperone activity in preventing heat‐induced citrate synthase (CS) from aggregation. As a result, both His‐HSCA2‐Myc and His‐HSCA2^G87D^‐Myc significantly repressed CS aggregation, and His‐HSCA2‐Myc displayed higher chaperone activity (Figure 4d).

*Ethylene response factor 9* (*ZmERF9*) is a candidate gene associated with phosphorus deficiency in a genome‐wide association study (GWAS). The highest associated sites included two single‐nucleotide polymorphisms (SNPs) leading to two nonadjacent SAASs, and one indel near the EAR domain, which have been reported to modulate transcriptional inhibitory activity (Ohta *et al*., 2001). We quantified the transcriptional inhibitory activity of ZmERF9 between two inbred lines DAN599 and CIMBL145 and detected that ZmERF9^DAN599^ had a significantly higher level of transcriptional inhibitory activity than ZmERF9^CIMBL145^ (Figure 4e). We speculated that the higher level of inhibitory activity of ZmERF9^DAN599^ might be caused by one of the associated SNPs that have been detected in the GWAS. PPVED was used to predict the effect of two SAASs on protein activities of ZmERF9. Although ZmERF9^R204Q^ and ZmERF9^T211S^ were predicted and experimentally validated to be neutral (the indel was also neutral; Table 5, Figure 4e), these results confirmed that the causal variants were not on the highest associated sites but somewhere having linkage disequilibrium (LD) with these loci. Collectively, the wet lab experiments validated the ability of PPVED with a 0.833 predictive accuracy.

## Discussion

The characterization of the effect of SAASs on protein function is of biological importance and can help provide a deeper understanding of the molecular basis of diseases and other complex traits (Kono *et al*., 2018; Kovalev *et al*., 2018; Wang *et al*., 2012). Plant science significantly lags behind human science in the development of databases for sharing variant information or tools for detecting functional genetic variants and predicting their effect on proteins or phenotypes (Amberger *et al*., 2014; Landrum *et al*., 2018; Nair and Vihinen, 2013; Schaafsma and Vihinen, 2015; Schaefer *et al*., 2012; Sherry *et al*., 2001; Stenson *et al*., 2017; Yip *et al*., 2010). Until now, there has been no well‐curated SAASs or prediction tools specifically designed for plants to characterize genomes for detecting substitutions that change protein function. In this study, we introduced a plant SAASs database containing 12 865 SAASs collected from multiple resources and presented PPVED as a machine learning‐based web service to predict the effect of SAASs on protein function. This study contributes to plant science in many aspects, namely, aiding discoveries of causal variants, providing application of machine learning in solving biological questions, and storing and organizing useful molecular polymorphisms into a plant‐specific database.

Huge amounts of SNPs were generated from whole‐genome sequencing and resequencing projects (Carlos *et al*., 2016; Hufford *et al*., 2021; Scheben *et al*., 2019; Tao *et al*., 2021; Zhao *et al*., 2018). However, most SNPs lack experimental‐level evidence to support their functionality, which is unsuitable for use as the training set to build the model in this study. Thus, numerous functional SAASs, as well as a set of simulated pseudoneutral SAASs, were manually curated for the development of PPVED. Several previous studies (Bromberg and Rost, 2007; Hecht *et al*., 2015; Kono *et al*., 2018; Kovalev *et al*., 2018) have simulated numerous neutral SAASs for predicting SAASs pathogenicity through computational methods; for example, ~65% (26 840 of 41 174) of neutral SAASs were obtained by simulation in the human SNAP database (Bromberg and Rost, 2007). Advances in prediction tools can facilitate accurate and high‐throughput screening of variants and accelerate the subsequent validation and annotation of variants in the future.

The implementation of machine learning in classification and prediction of genomic variants has been advanced in recent years and various supervised algorithms have been used to predict the functional impact of these variants (Adzhubei *et al*., 2010; Capriotti *et al*., 2006; Hecht *et al*., 2015; Niroula *et al*., 2015; Pejaver *et al*., 2020; Quang *et al*., 2015; Wang *et al*., 2012). Indeed, these algorithms have improved prediction accuracy and generalization ability when compared with the classical method of sequence conservation. Although a set of distinct algorithms have been used to learn models for prediction, no single algorithm consistently outperforms others and there is no consensus on which algorithm is appropriate in predicting the functional effect of SAASs. In this study, we presented three types of machine learning algorithms (parameter‐based, tree‐based, and ensemble) and compared their global performance in terms of SEN, SPE, PRE, ACC, MCC, and AUC. Our results indicate that tree‐based algorithms (such as RF and XGBoost) are more suitable than parameter‐based algorithms (such as SVM and FFNN) (Table 1 and Table 2). Consistently, more existing software has applied tree‐based algorithms, such as FunSAV (Wang *et al*., 2012), PON‐P2 (Niroula *et al*., 2015), REVEL (Ioannidis *et al*., 2016), ClinPred (Alirezaie *et al*., 2018), InMeRF (Takeda *et al*., 2020), and MISTIC (Chennen *et al*., 2020). Furthermore, we also found that ensemble models cannot lead to better prediction results compared with individual models.

Model assessment is necessary and can prove the generalization ability and applicability of the model. In this study, three various assessments were performed to verify the excellence of PPVED. First, we compared PPVED with six previously reported software in the benchmark and independent datasets, respectively (Table 3, Table 4, and Figure 3). The results indicated that PPVED had the best performance and was robust. However, most of the software had certain defects, such as the high false‐positive rate of SIFT and the high negative rate of MutPred2. These results are consistent with previous views that the transfer of knowledge of distant cross‐species has certain limitations (Feiz *et al*., 2009; Kono *et al*., 2018). Second, we further validated PPVED using 314 newly collected functional SAASs and 1515 neutral SAASs. The predictive accuracy was consistent with the benchmark or independent datasets and reflected the generalization ability of PPVED. Finally, we validated the predictive ability of PPVED through wet lab experiments on three proteins (Table 5, Figure 4); the results were almost completely consistent with the observations, suggesting the applicability of PPVED for detecting functional genetic variants in plants.

## Methods

### Dataset

We manually curated a set of plant SAAS datasets, which contained two subcategories: functional and neutral SAASs. The functional SAASs were represented by physiological or morphological changes (Kono *et al*., 2018). These SAASs were obtained from three sources. The first was UniProt/Swiss‐Prot (https://www.uniprot.org, release 2019_10). We selected all complete proteins classified as ‘Viridiplantae’ with mutagenesis information from the UniProt/Swiss‐Prot database and then manually retrieved the mutagenesis annotations of these proteins. We excluded non‐SAASs, including insertions, deletions, and multi‐amino acid substitutions, and finally obtained 5751 SAASs. Of these SAASs, 4964 were functional and 787 were neutral. The second source of SAASs was NCBI/PubMed (https://pubmed.ncbi.nlm.nih.gov). We retrieved literatures that may be associated with plant SAASs from the NCBI/PubMed database using a set of preset keywords (see Table S4). We obtained 2468 SAASs, of which 2067 were functional and 401 were neutral. The third source of SAASs was simulation calculation. Considering the unbalanced proportions of the above subcategories, we referred to previous computational methods; that is, we simulated a set of pseudoneutral SAASs based on multiple sequence alignments (MSAs) (Kovalev *et al*., 2018). First, we downloaded all complete proteins classified as ‘Viridiplantae’ in the UniProt/Swiss‐Prot (library SP) and UniProt/TrEMBL (library TR) databases. Next, we aligned each protein in library SP with library SP+TR using BLASTP (Altschul *et al*., 1990). We retained the hit proteins with sequence identity of ≥ 95%. Then, the query protein was further aligned with the hit proteins using Clustal Omega (Sievers and Higgins, 2014), and the SAASs were filtered according to the following strict rules: MSAs contained no less than three sequences; only two kinds of amino acids could appear in each column; only one substitution could appear in the cluster consisting of five amino acids; and only one substitution could appear in the pairwise alignment results (a detailed explanation of the filtering rules is shown in Figure S3). Finally, the Needleman Wunsch algorithm (Needleman and Wunsch, 1970) was used to further filter SAASs that were repeatedly recorded. The threshold of sequence identity was ≥ 95%. Finally, we obtained 5391 pseudoneutral SAASs.

We integrated the SAASs collected from the above three sources and excluded SAASs with conflicting labels (functional or neutral). In summary, we obtained a total of 12 865 plant SAASs, of which 6367 were functional and 6498 were neutral. These SAASs had a roughly balanced ratio of 1:1 and were evenly distributed in 6172 proteins. We randomly selected 5094 functional and 5198 neutral SAASs as the benchmark dataset (80% of the dataset) to build and tune the model. The remaining SAASs, comprising 1273 functional and 1300 neutral SAASs, were used for the independent dataset (20% of the dataset) to validate the generalization ability of the model.

To further validate the model, 314 newly plant functional SAASs from the UniProt/Swiss‐Prot (release 2020_05) database were collected according to the above method. All datasets used in this study can be downloaded from http://www.ppved.org.cn. Notably, for each SAAS, we recorded the following detailed information: source, organism, protein accession number, protein sequence source database, protein sequence, wildtype amino acid, mutant amino acid, mutation position, PMID of supporting literature, supporting experimental evidence, and label.

### Feature extraction

We comprehensively collected the features that characterize SAASs, and these features were roughly divided into the following five categories

#### Sequence evolutionary features

It is reported that evolutionarily conserved positions are frequently associated with disease‐related mutations in humans (Miller and Sudhir, 2001; Wang *et al*., 2012), and some studies have applied evolutionary information to predict SAAS pathogenicity (Ng and Henikoff, 2001; Wang *et al*., 2012). Therefore, we referred to a previous computational method (Ng and Henikoff, 2001); PSI‐BLAST (Altschul *et al*., 1997) was used in generating a position‐specific score matrix (PSSM) by aligning the protein with the above library SP+TR. We collected the following five features: (1) position‐specific score of wildtype amino acid (PSSM_FROM); (2) position‐specific score of mutant amino acid (PSSM_TO); (3) absolute value of the difference in the position‐specific score (PSSM_CHANGE); (4) substitution frequency of SAASs in the alignment results (SFM); and (5) conservation score of mutation position (CON_SCORE). The formula was as follows:

$${\mathrm{CON}\_\mathrm{SCORE}}_{i}=\;‐\sum_{j=1}^{n=20}P_{i,j}\log_{2}P_{i,j}$$

where *P_i,j_* is the frequency of amino acid *j* at position *i*.

#### Physicochemical features

The latest version of the AAindex database [https://www.genome.jp/aaindex, v9.2] (Kawashima *et al*., 2008) stores >700 kinds of physicochemical information of amino acids and contains a total of three subdatabases (AAindex1, AAindex2, and AAindex3). Some studies have also applied AAindex to predict SAAS pathogenicity (Chennen *et al*., 2020; Niroula *et al*., 2015). Therefore, we collected all physicochemical information in AAindex and eliminated the entries comprising missing or conflicting comments. Moreover, to ensure the accuracy of the information, we also eliminated the entries annotated as asymmetric matrices from AAindex2 and AAindex3. Finally, we collected 631 physicochemical features in total, and all features were named using the original accession number in the AAindex.

#### Database annotated features

The UniProt/Swiss‐Prot database stores numerous protein annotations, such as protein structures, functions, and post‐translational modifications. Some studies have applied these annotations to make predictions (Niroula *et al*., 2015; Wang *et al*., 2012). We collected 25 annotations in total, which belonged to five categories: (1) Function, which contained BINDING, ACT_SITE, SITE, METAL, DNA_BIND, NP_BIND, CA_BIND, and EC; (2) PTM/Processing, which contained LIPID, DISULFID, MOD_RES, CARBOHYD, PROPEP, SIGNAL, and TRANSIT; (3) Subcellular location, which contained TOPO_DOM, TRANSMEM, and INTRAMEM; (4) Family&Domains, which contained MOTIF, DOMAIN, REGION, and ZN_FING; and (5) Structure, which contained HELIX, STRAND, and TURN. We collected more annotations compared with previous studies. Next, we used these annotations according to the following rules: (i) we aligned the protein to the UniProt/Swiss‐Prot database using BLASTP and found the best hit protein in the alignment results; (ii) we corrected the position of SAAS in the hit protein according to the alignment results and calculated the shortest relative distance (SRD) between the SAAS and the annotations on the hit protein; (iii) as some proteins lack some annotations, we merged all annotations in each category (except EC) to reduce the effect of the lack of annotations. Finally, we collected 31 database annotated features in total, of which 25 were individual annotations and 6 were merged annotations. For individual annotations, the original abbreviation was directly used for the names of the features; for the merged annotations, the names of the features were as follows: FUNCTION_MERGE, PTM_MERGE, SUBLOC_MERGE, FAMILY_MERGE, STRUCTURE1_MERGE, and STRUCTURE2_MERGE. The following formulas were applied for these annotations:

$${\text{RD}}_{\mathrm{ij}}=\left\{\begin{array}{cc} \frac{\left|\text{position}‐{\text{annotation}}_{\mathrm{ij}}\right|}{\text{length}}, & \text{the ith annotation exists}\\ 1, & \;\text{the ith annotation does not exist} \end{array}\right.$$

$${\mathrm{SRD}}_{i}=\min\left\{{\mathrm{RD}}_{i1},{\;\mathrm{RD}}_{i2},\cdots ,{\;\mathrm{RD}}_{\mathrm{in}}\right\}$$

where position is the position of SAAS, annotation*_ij_* is the *j*th annotation position of the *i*th annotation, and length is the length of the hit protein.

#### Predicted features

Some studies have revealed that other information, such as protein secondary structure, solvent accessibility, and enzyme function, is useful for SAAS prediction (Saunders and Baker, 2002; Gao *et al*., 2015); however, these data are difficult to obtain for plant proteins. Therefore, we extensively used software to predict this information based on protein sequences, mainly including the prediction of the following: (1) secondary structure and relative solvent accessibility of proteins using SCRATCH v1.2 (Magnan and Baldi, 2014); (2) disordered regions of proteins using DISOPRED v3.16 (Ward *et al*., 2004); (3) protein aggregation using TANGO v2.3.1 (Fernandez‐Escamilla *et al*., 2004); (4) half‐sphere exposure of proteins using HSEpred (Song *et al*., 2008); (5) disulphide bonds of proteins using DIpro v2.0 (Cheng *et al*., 2010); (6) protein domains using DOMpro v1.0 (Cheng *et al*., 2006); (7) transmembrane helix and signal peptide of proteins using MEMSAT‐SVM v1.3 (Nugent *et al*., 2010); (8) nuclear localization signal of proteins using NLStradamus v1.8 (Ba *et al*., 2009); (9) phosphorylation sites of proteins using NetPhos v3.1 (Blom *et al*., 2004); (10) ubiquitination sites of proteins using UbPred (Radivojac *et al*., 2010); (11) O‐glycosylation sites of protein using NetOGlyc v3.1 (Steentoft *et al*., 2013); (12) N‐glycosylation sites of proteins using NetNGlyc v1.0 (Gupta and Brunak, 2002); (13) protein stability changes upon single point mutation using I‐Mutant v2.0.7 (Emidio *et al*., 2005); (14) solubility of proteins using SOLpro (Magnan *et al*., 2009); (15) enzyme function of proteins using EFICAz v2.5.1 (Kumar and Skolnick, 2012); and (16) subcellular localization of proteins using LocTree3 (Tatyana *et al*., 2014). Finally, we collected a total of 328 predicted features.

#### Coevolutionary features

Coevolutionary features can be used to identify important coevolutionary residues and have been applied in previous studies (Wang *et al*., 2012), which are more likely to be rich in disease‐related mutations (Kowarsch *et al*., 2010). We used mutual information (MI) to characterize coevolution using the following computational methods: (i) we aligned the protein to the above library SP+TR using BLASTP; (ii) to ensure MSAs will be large and diverse (Simonetti *et al*., 2013), we selected N (100–1,000, interval of 100) hit sequences for further multiple alignments with the query protein using Clustal Omega; and (iii) we calculated the MI between the wildtype amino acid of SAAS and the amino acid of adjacent positions −4, −3, −2, −1, +1, +2, +3, and +4. Moreover, the minimum, maximum, and mean MI in these eight positions were calculated. We also considered whether there is a gap in the alignment results. Finally, a total of 220 coevolutionary features were collected. The calculation formula for MI was as follows:

$$MI\left(i,j\right)=\sum_{a,b}P\left(a_{i},b_{j}\right)\log_{2}\frac{P\left(a_{i},\;b_{j}\right)}{P\left(a_{i}\right)P\left(b_{j}\right)}$$

where *P(a_i_,b_j_)* is the frequency of amino acid *a* at position *i* and amino acid *b* at position *j* in the same sequence, *P(a_i_)* is the frequency of amino acid *a* at position *i*, and *P(b_j_)* is the frequency of amino acid *b* at position *j*.

In summary, we collected 1215 features that characterize SAASs and standardized these features using the z‐score method. The standardization rules were as follows: (i) we standardized the benchmark dataset and recorded the parameters of each feature, including the mean and standard deviation (SD); and (ii) we applied the parameters to the independent dataset, thereby standardizing the independent dataset. The calculation formula for the z‐score method was as follows:

$$x_{\mathrm{ij}}^{z‐\text{score}}=\frac{x_{\mathrm{ij}}‐{\bar{x}}_{i}}{\sigma_{i}}$$

where *x_ij_* is the *j*th value of the *i*th feature, ${\bar{x}}_{i}$ is the mean of the *i*th feature, and *σ_i_* is the SD of the *i*th feature.

### Feature selection

**Figure 5:**
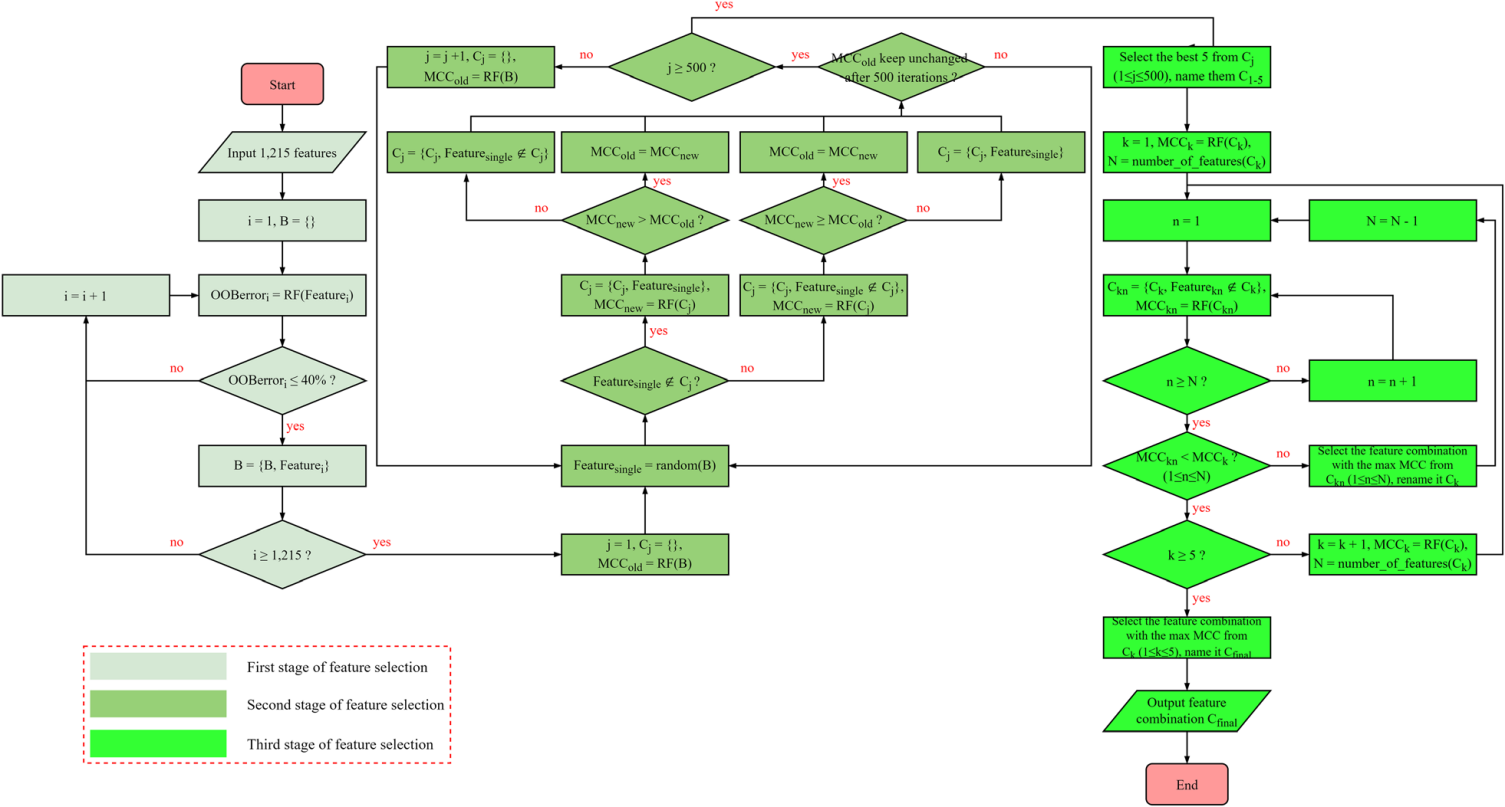
The three‐step feature selection pipeline. We used individual feature to build the RF models, and then used the out‐of‐bag (OOB) error of the RF models to exclude (OOB error ≤ 40%) meaningless features in the first stage of feature selection. We further repeated the heuristic‐based genetic algorithm (GA) for 500 times in the second stage of feature selection. The termination condition of GA was that the performance kept unchanged after 500 iterations. Finally, we performed backward feature selection on the five optimal feature combinations produced by GA in the third stage of feature selection.

Feature selection can reduce the dimensions of features and avoid model overfitting. However, it is extremely challenging to select important and informative features from among numerous features. Here, we designed a three‐step feature selection pipeline to select the optimal feature combinations for the prediction of SAASs from the above 1215 features. In this pipeline, we used the RF with default hyperparameters provided by the randomForest v4.6‐14 package to build the models. Notably, all RF models were built by repeating stratified fivefold cross‐validation 10 times. The details of the pipeline are shown in Figure 5, and briefly described below. We used the mean MCC of 50 models to evaluate the performance of the RF models, as follows:

$$\mathrm{MCC}=\frac{\mathrm{TP}\times \mathrm{TN}‐\mathrm{FP}\times \mathrm{FN}}{\sqrt{\left(\mathrm{TP}+\mathrm{FN}\right)\times \left(\mathrm{TP}+\mathrm{FP}\right)\times \left(\mathrm{TN}+\mathrm{FN}\right)\times \left(\mathrm{TN}+\mathrm{FP}\right)}}$$

where TP is the number of true positives, TN is the number of true negatives, FP is the number of false positives, and FN is the number of false negatives.

#### First stage of feature selection

We used individual feature to build the RF models, thereby maintaining consistency between the number of models and features. Then, we used the out‐of‐bag (OOB) error of the RF models to exclude meaningless features, and the features (*B* in Figure 5) of OOB error ≤ 40% were retained (see Table S5).

#### Second stage of feature selection

A heuristic‐based genetic algorithm (GA) was used for feature selection. In each iteration, we randomly selected a feature *Feature_single_* from *B* and decided whether to include or exclude it from *C_j_* according to the state of *Feature_single_*. The above steps were repeated until the performance remained unchanged after 500 iterations. Notably, the GA produced a local optimal solution because of its strong randomness. To improve the possibility of selecting the optimal feature combinations, we repeated the GA 500 times, yielding 500 sets of feature combinations (*C_1_, C_2_, …, C_500_*).

#### Third stage of feature selection

Finally, a backward feature selection algorithm (BFSA) was used for feature selection. Here, we only performed BFSA on the five optimal feature combinations (*C*_1_ *, C*_2_ *, C*_3_, *C*_4_, *C*_5_) produced by GA, from which an optimal feature combination (*C*_final_) was selected.

### Model building

Previous studies have used various machine learning algorithms, such as RF (Niroula *et al*., 2015; Wang *et al*., 2012), SVM (Capriotti *et al*., 2006), and FFNN (Hecht *et al*., 2015; Pejaver *et al*., 2020). Therefore, we also used four machine learning algorithms, namely, RF provided by the randomForest v4.6‐14 package, XGBoost provided by the xgboost v0.90.0.2 package, SVM provided by the e1071 v1.7‐4 package, and FFNN provided by the neuralnet v1.44.2 package. We tuned their hyperparameters by repeating stratified fivefold cross‐validation 10 times. For RF, we tuned three hyperparameters, including ntree, mtry, and nodesize; for XGBoost, we tuned nine hyperparameters, including nrounds, max_depth, min_child_weight, gamma, subsample, colsample_bytree, alpha, lambda, and eta; for SVM, we tuned three hyperparameters, including kernel, gamma, and cost; and for FFNN, we only considered a single hidden layer network (Pejaver *et al*., 2020) with a backpropagation algorithm and tuned three hyperparameters, including act.fct, hidden, and learningrate. Considering the task complexity and runtime efficiency of the prediction of SAASs, we only considered a single hidden layer network, which is similar to previous studies (Hecht *et al*., 2015; Pejaver *et al*., 2020). After determining the optimal hyperparameters of each algorithm, we trained 1,000 models by repeating stratified fivefold cross‐validation 10 times; thus, the performance of each model was represented by the mean of 50 submodels. We selected one optimal model from these 1000 models.

Additionally, to validate the complementarity of the above four algorithms for the prediction of SAASs and to further improve model performance, we also built 11 ensemble models (logistic regression [LR] with *glm* function in R) using the stacking method based on the four algorithms. In summary, we built a total of 15 machine learning models, of which 4 were individual models and 11 (*C*2 4+*C*3 4+*C*4 4) were ensemble models.

### Performance evaluation

We used the SEN, SPE, PRE, ACC, MCC, and AUC as indicators to systematically evaluate the performance of the models from different aspects. AUC was calculated using the pROC v1.16.2 package (Robin *et al*., 2011), and the calculation formulas of other indicators were as follows:

$$\mathrm{SEN}=\frac{\mathrm{TP}}{\mathrm{TP}+\mathrm{FN}}$$

$$\mathrm{SPE}=\frac{\mathrm{TN}}{\mathrm{TN}+\mathrm{FP}}$$

$$\mathrm{PRE}=\frac{\mathrm{TP}}{\mathrm{TP}+\mathrm{FP}}$$

$$\mathrm{ACC}=\frac{\mathrm{TP}+\mathrm{TN}}{\mathrm{TP}+\mathrm{TN}+\mathrm{FP}+\mathrm{FN}}$$

where TP is the number of true positives, TN is the number of true negatives, FP is the number of false positives, and FN is the number of false negatives.

### Performance comparison

As it is quite difficult to find a prediction tool specific to plant SAASs, we selected six types of popular existing software that were developed based on human SAASs to compare their performance with our methods. Performance assessment of each existing software and PPVED used the same benchmark dataset and independent dataset. Three types of software developed based on sequence conservation (SIFT, PROVEAN, and PANTHER‐PSEP), and three that were developed based on machine learning (PhD‐SNP, PolyPhen‐2, and MutPred2) were used. The two models provided by PolyPhen‐2, HumDiv, and HumVar were considered. No software developed based on hybrid methods is applicable to plants.

### Online website

To ensure that our proposed models can be utilized, a user‐friendly online website was developed. We used Apache as the web server and Perl as the backend language to write the common gateway interface. The website operates on a 64‐bit CentOS Linux server with a basic configuration of eight cores and 32G. The homepage of the website is http://www.ppved.org.cn.

**Figure 6:**
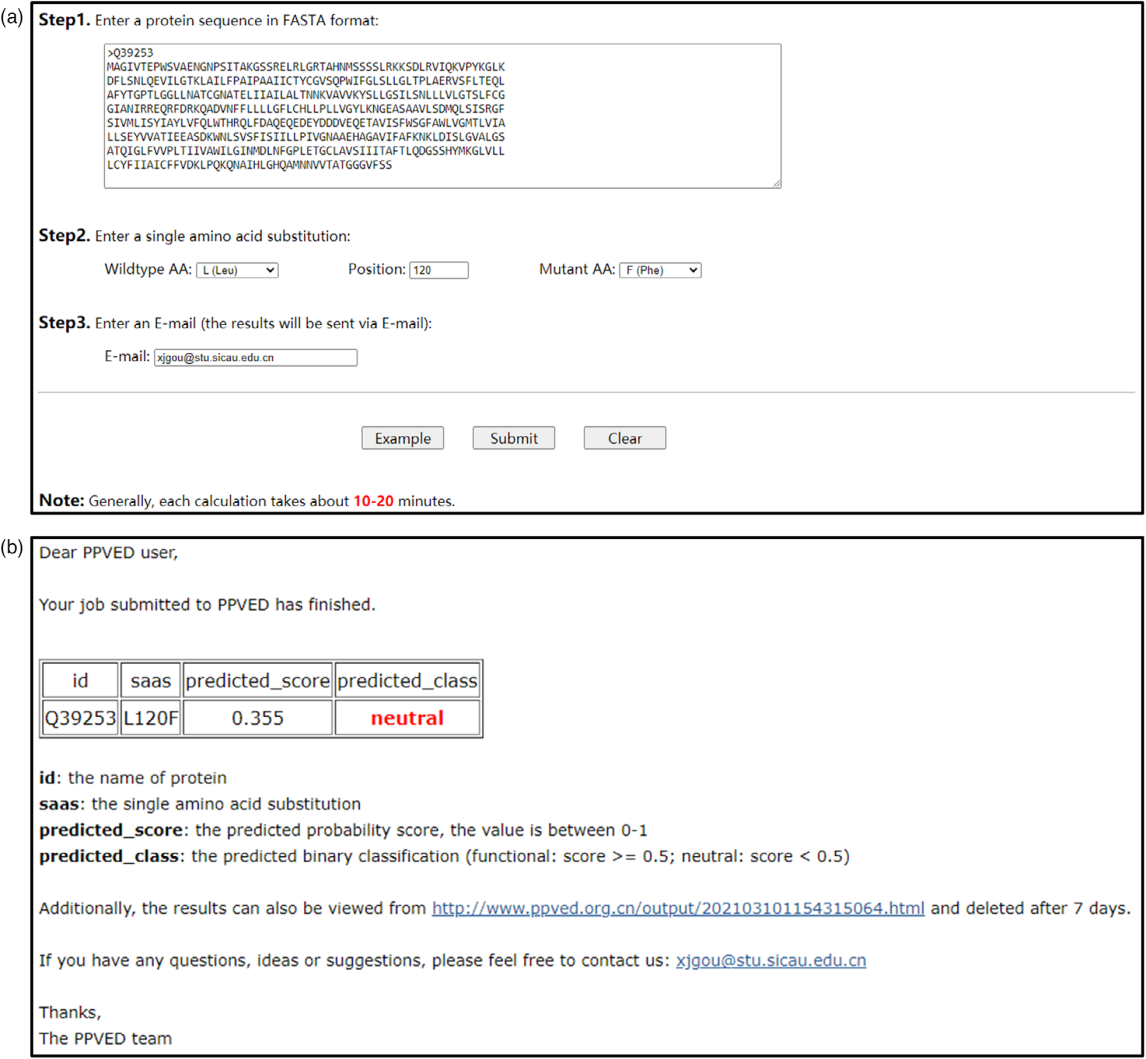
Online service of PPVED. (a) User interface of PPVED. The users need to fill in three kinds of information, including protein sequence, amino acid substitution, and the email to receive the prediction results. (b) Output example of PPVED. PPVED will output four kinds of information, including the submitted protein id, the submitted amino acid substitution, the predicted probability score (the value is between 0–1), and the predicted binary classification (functional: score ≥ 0.5; neutral: score <0.5).

The user interface is shown in Figure 6a; users need to provide three pieces of information to receive the prediction results: protein sequence, amino acid substitution, and email. The results included the predicted score and binary classification, and the classification is predicted to be functional when the predicted score is ≥ 0.5; the classification is predicted to be neutral when the predicted score is <0.5 (an explanation of why 0.5 is used as the threshold is shown in the Figure S4). Generally, the results are sent within 10–20 min, as shown in Figure 6b. Notably, the website only supports the submission of one SAAS at a time; thus, if users need to make numerous predictions, they are encouraged to download the local installation package provided by the website.

### Plasmid construction and plant transformation

All constructs and primers used in this study are listed in Table S6. Briefly, for genetic complementation, the genomic sequences that encode HSCA2^G87D^, ISU1^T55 M^, ISU1^G106D^, and ISU1^A143T^ were amplified and cloned from corresponding suppressor mutants. All complementation constructs were based on the binary vector pCAMBIA1300. *Arabidopsis* plants were transformed with the agrobacteria‐mediated floral dipping method (Clough and Bent, 1998). The transgenic plants were screened on a hygromycin B‐containing Murashige and Skoog medium. The integration of the transgene was confirmed by polymerase chain reaction (PCR).

### Protein expression and purification from *E. coli*

His_6_‐tagged protein expression and purification from *E. coil* were carried out as described previously (Leaden *et al*., 2014). Briefly, BL21 (DE3) bacterial strains with respective constructs were cultured in LB liquid medium at 37 °C to OD_600nm_ ≈ 0.5 and then induced with IPTG at a final concentration of 1 mm for 6 h at 28 °C. Cells were harvested, resuspended in buffer A (20 mm Tris–HCl, 200 mm NaCl, 30 mm imidazole, and 1 mm phenylmethylsulfonyl fluoride [PMSF], pH 7.4), and then disrupted by sonication. The suspensions were centrifuged at 10,000 × *g* for 15 min at 4°C. The supernatants of the His_6_‐tagged proteins obtained were incubated with 500 μL Ni Sepharose (GE Healthcare 17‐5318‐06, U.S.A.) and then washed twice with buffer A. The recombinant proteins were eluted with buffer B (500 mm imidazole in buffer A). The eluents were further applied to size exclusion chromatography with the Superdex 75 or Superdex 200 column with ÄKTA Purifier 10 FPLC system (GE Healthcare).

### In vitro chaperone activity assay

All the tested proteins and citrate synthase (CS) (Sigma, C3260, USA) were dialyzed in 20 mm HEPES‐KOH, pH 7.5, 150 mm KCl, and 10 mm MgCl_2_ before being used for the heat‐induced aggregation assay. CS (500 nm) was prepared in a final volume of 150 mL 20 mm HEPES‐KOH (pH 7.5) and 2.8 mm β‐mercaptoethanol with different amounts of tested proteins. The mixtures were loaded onto a 96‐well microplate and heated at 45°C. Light scattering at 340 nm was monitored at 45°C in a Synergy 4 spectrophotometer (BioTek) for 90 min. Control measurements were performed with commercial bovine serum albumin (BSA).

### Transcriptional inhibitory activity test

Different allelotypes of *ZmERF9* were fused with the *Gal4‐AD* sequence. Then, the fused fragments were cloned into pGBKT7 and further fused with the *Gal4‐BD* sequence. *Gal4‐AD* was cloned into pGBKT7 to be fused with the *Gal4‐BD* sequence, resulting in the complete *Gal4*,which was used as a control. The transformation was conducted according to the manual of Yeast Protocols Handbook (Clontech). Primers and constructions are listed in Table S6. The combination of pGBKT7‐53 and pGADT7‐T was used as a positive control (+). The combination of pGBKT7‐Lam and pGADT7‐T was used as a negative control (−).

## Conflicts of interest

The authors declare that they have no competing interests.

## Author contributions

Yanli Lu and Yaxi Liu conceived the study and participated in its design and coordination; Xiangjian Gou, Haoran Shi, and Qi Wang analysed the data and developed the PPVED tool; Xuanjun Feng and Rongqian Xie performed the experiments; Xiangjian Gou, Hongxiang Li, Banglie Yang, and Lixue Chen collected the dataset; Xiangjian Gou drafted the manuscript; Xiangjian Gou, Tingting Guo, Xuanjun Feng, Yaxi Liu, and Yanli Lu revised the manuscript. All the authors read and approved of the final version of the manuscript.

## Supporting information


**Figure S1**. Performance comparison between 48 features‐based models (after feature selection) and 1,215 features‐based models (before feature selection).
**Figure S2**. Model performance when removed a class of features and used the remaining features to train the models.
**Figure S3**. The filtering rules of simulation‐based single amino acid substitutions (SAASs) used in this study.
**Figure S4**. Threshold of predicted score for distinguishing functional and neutral single amino acid substitutions.

**Table S1**. The 48 features finally selected.

**Table S2**. Prediction results of 314 newly collected SAASs from the UniProt/Swiss‐Prot database.

**Table S3**. Prediction results of 1,515 neutral SAASs from the published literature.

**Table S4**. Preset keywords for retrieving literatures from the NCBI/PubMed database.

**Table S5**. Out‐of‐bag error of each feature in the first stage of feature selection.

**Table S6**. Plasmid constructs and primers used in this study.
