# Supplementary material for: PPVED: A machine learning tool for predicting the effect of single amino acid substitution on protein function in plants
Source: Plant Biotechnol J. 2022 Apr 27;20(7):1417–31. doi: 10.1111/pbi.13823 (PMC9241370; doi:10.1111/pbi.13823)
Supplement: Supplementary file 1 — Figure S1. Performance comparison between 48 features‐based models (after feature selection) and 1,215 features‐based models (before feature selection). Figure S2. Model performance when removed a class of features and used the remaining features to train the models. Figure S3. The filtering rules of simulation‐based single amino acid substitutions (SAASs) used in this study. Figure S4. Threshold of predicted score for distinguishing functional and neutral single amino acid substitutions. [file PBI-20-1417-s005.pdf]

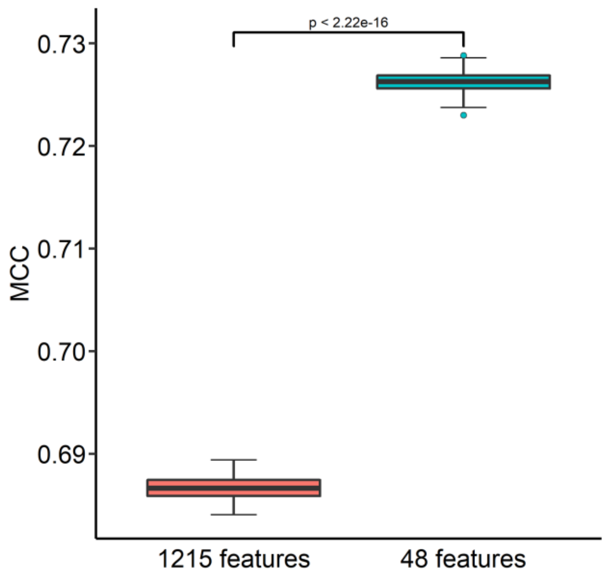

Figure S1 Performance comparison between 48 features-based models (after feature selection) and 1,215 features-based models (before feature selection). The random forest with default hyperparameters provided by the randomForest v4.6-14 package was used to train the models, and the performance was represented by Matthew's correlation coefficient (MCC). Each feature combination (48 features or 1,215 features) was repeatedly used to trained the models for 100 times. The results indicated that the three-step feature selection pipeline proposed in this study was useful, and the model performance after feature selection was significantly higher than that before feature selection.

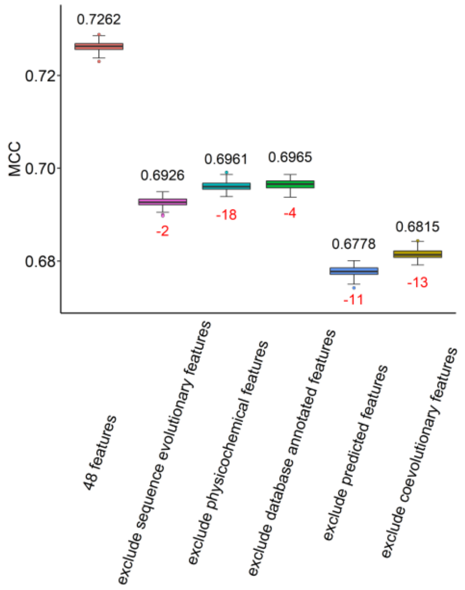

Figure S2 Model performance when removed a class of features and used the remaining features to train the models. The random forest with default hyperparameters provided by the randomForest v4.6-14 package was used to train the models, and the performance was represented by Matthew's correlation coefficient (MCC). Each feature combination was repeatedly used to trained the models for 100 times. The number on the upper of the box plot represented the average MCC, and the number on the lower of the box plot represented the number of features removed. The *t*-test indicated that the reduction in performance was significant upon removing a class of features compared to complete 48 features ($P<0.01$).

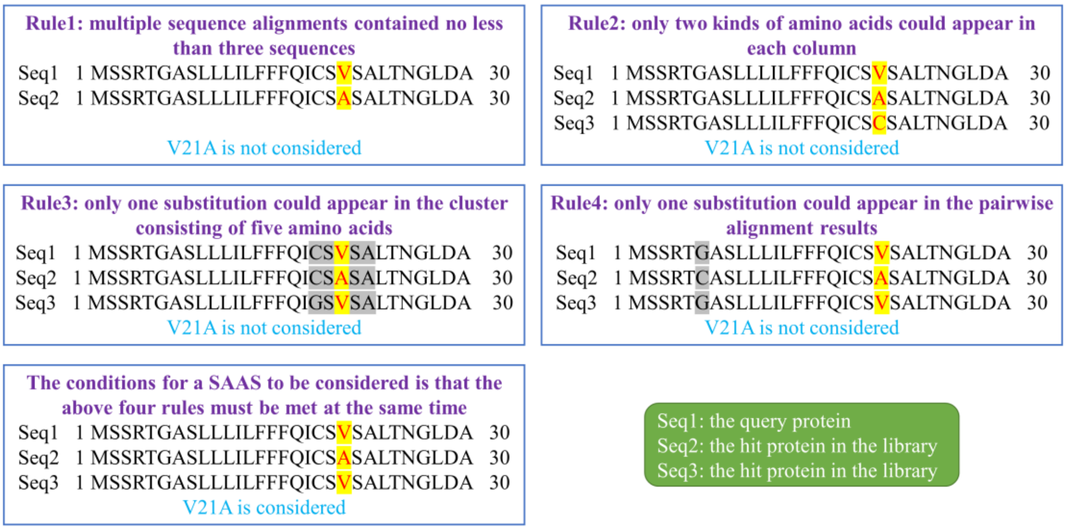

Figure S3 The filtering rules of simulation-based single amino acid substitutions (SAASs) used in this study. The four rules included (1) multiple sequence alignments contained no less than three sequences; (2) only two kinds of amino acids could appear in each column; (3) only one substitution could appear in the cluster consisting of five amino acids; and (4) only one substitution could appear in the pairwise alignment results. The conditions for a SAAS (e.g. V21A at yellow highlighted area) to be considered neutral is that the above four rules must be met at the same time.

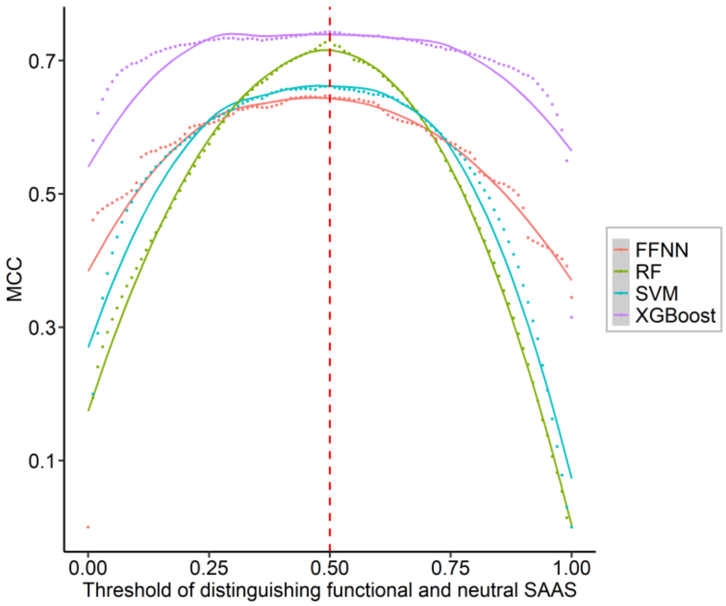

Figure S4 Threshold of predicted score for distinguishing functional and neutral single amino acid substitutions. The vertical red dotted line indicated that the threshold is 0.5. MCC, Matthew's correlation coefficient; RF, random forest; XGBoost, extreme gradient boosting; SVM, support vector machine; FFNN, feedforward neural network. The results indicated that each algorithm had the largest MCC when the threshold is 0.5.
